# Novel Perfused Compression Bioreactor System as an *in vitro* Model to Investigate Fracture Healing

**DOI:** 10.3389/fbioe.2015.00010

**Published:** 2015-02-02

**Authors:** Waldemar Hoffmann, Sandra Feliciano, Ivan Martin, Michael de Wild, David Wendt

**Affiliations:** ^1^Department of Biomedicine, University Hospital Basel, Basel, Switzerland; ^2^Department of Surgery, University Hospital Basel, Basel, Switzerland; ^3^School of Life Sciences, Institute for Medical and Analytical Technologies, University of Applied Sciences Northwestern Switzerland, Muttenz, Switzerland

**Keywords:** bioreactor, *in vitro* model, mechanical loading, hypertrophy, fracture healing

## Abstract

Secondary bone fracture healing is a physiological process that leads to functional tissue regeneration via endochondral bone formation. *In vivo* studies have demonstrated that early mobilization and the application of mechanical loads enhances the process of fracture healing. However, the influence of specific mechanical stimuli and particular effects during specific phases of fracture healing remain to be elucidated. In this work, we have developed and provided proof-of-concept of an *in vitro* human organotypic model of physiological loading of a cartilage callus, based on a novel perfused compression bioreactor (PCB) system. We then used the fracture callus model to investigate the regulatory role of dynamic mechanical loading. Our findings provide a proof-of-principle that dynamic mechanical loading applied by the PCB can enhance the maturation process of mesenchymal stromal cells toward late hypertrophic chondrocytes and the mineralization of the deposited extracellular matrix. The PCB provides a promising tool to study fracture healing and for the *in vitro* assessment of alternative fracture treatments based on engineered tissue grafts or pharmaceutical compounds, allowing for the reduction of animal experiments.

## Introduction

Bone fracture healing is a natural, physiological process leading to functional tissue regeneration through a highly orchestrated sequence (Gerstenfeld et al., 2003; Behonick et al., 2007; Marsell and Einhorn, 2011). Primary fracture healing occurs within stable fracture sites when there is direct contact between the fracture ends, re-establishing the anatomically correct and biomechanically competent lamellar bone structure (Marsell and Einhorn, 2011). However, in the majority of cases a gap is present at the fracture site, and therefore indirect or secondary fracture healing occurs.

Secondary fracture healing, a process recapitulating the process of endochondral bone formation, is divided into four main phases: hemorrhage and inflammation, soft callus formation, hard callus formation, and callus remodeling (Sfeir et al., 2005; Schindeler et al., 2008). Following initial hemorrhage and inflammation, a key step during secondary fracture healing is the formation of a soft fracture callus, consisting of cartilaginous extracellular matrix, chondrocytes, and fibroblasts. It provides mechanical support to the fracture and serves as a template for subsequent remodeling into a bony callus (Gerstenfeld et al., 2003; Sfeir et al., 2005; Schindeler et al., 2008). During the subsequent phase of hard callus formation, a mineralized cartilaginous template is gradually replaced with unordered woven bone matrix. The callus becomes vascularized, increasing the oxygen tension, and fostering maturation of osteoblasts (Sfeir et al., 2005; Schindeler et al., 2008). In the final phase, the woven bone is fully remodeled toward cortical and/or trabecular bone in a spatially and temporally choreographed manner (Sfeir et al., 2005; Schindeler et al., 2008).

*In vivo* models have demonstrated that mechanical stimulation of fractures can improve the secondary fracture healing process and/or alter the biological pathways involved (Rand et al., 1981; Goodship and Kenwright, 1985; Aro et al., 1991; Claes et al., 1997; Park et al., 1998; Rubin et al., 2001; Chao and Inoue, 2003). However, due to the multitude of parameters that play a role in the mechanical environment of the fracture site, these *in vivo* studies did not allow for a systematic study of specific mechanical stimuli nor their influence during the different phases of fracture healing (Gerstenfeld et al., 2003; Schindeler et al., 2008).

As an alternative, *in vitro* model systems facilitate a methodical approach to study the impact of the mechanical stimuli during distinct phases of secondary fracture healing in a controlled manner. However, *in vitro* models have previously been limited to applying mechanical loads on cartilaginous tissues in order to develop more functional tissues or to study the impact of different loading regimes on chondrogenesis (Démarteau et al., 2003; Ballyns and Bonassar, 2010; Sun et al., 2010; Puetzer et al., 2012), but the effect of mechanical loading during the process of hypertrophic cartilage formation and remodeling, critical in fracture healing, has not yet been studied.

Here, were propose an *in vitro* model based on a perfused compression bioreactor (PCB) system to: (i) apply physiological strain/loads, (ii) perfuse a construct allowing for mass transport and simulation of vascularization, and (iii) compress rigid load-bearing scaffolds in a physiological manner. The application of dynamic mechanical loading was validated for both collagen-based and nickel–titanium (NiTi) based tissue constructs, highlighting the broad operational range of the system including the compressive strength (100–200 MPa) of bone (Keaveny et al., 2004; Weiner and Wagner, 1998). In a proof-of-concept study, we hypothesized that physiological compressive loading applied during hypertrophy enhances extracellular matrix mineralization of cartilaginous constructs and triggers the maturation process of MSC toward late hypertrophic chondrocytes.

## Materials and Methods

### Perfused compression bioreactor system

**Figure 1:**
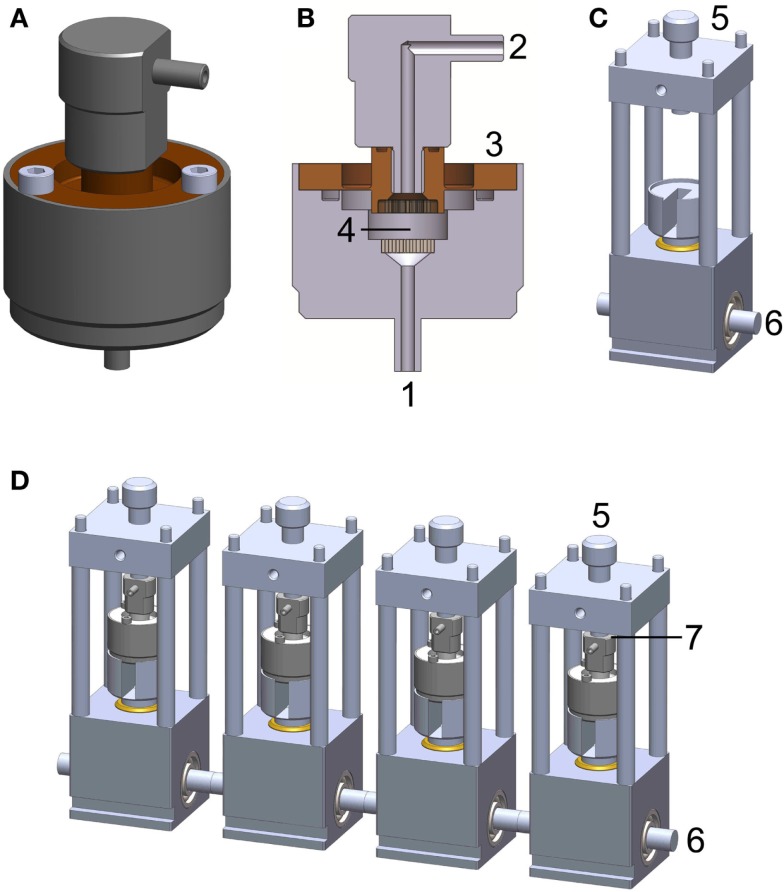
**Perfused compression bioreactor (PCB)**. **(A)** Bioreactor chamber holding the scaffold in place and ensuring hermetic sealing as well as force transmission toward the cell loaded construct. **(B)** Cross section of bioreactor chamber indicating medium inlets/outlets (1 and 2), flexible force transmitting disk (3), and intended space for scaffold/construct placement (4). **(C)** Power transmission rack including cam-shaft (6), which moves the plunger in order to apply a sinusoidal compression pattern onto the bioreactor chamber. The chamber is held in place with a pre-load screw (5) allowing for defined loading regimes. **(D)** A complete PCB system comprises four bioreactor chambers, four force transmission devices, and four force sensors (placed at position 7, not shown).

The PCB system consists of two main components: the bioreactor chamber (Figures 1A,B) and the power transmission rack (Figure 1C). A detailed description of the bioreactor system can be found in Hoffmann et al. (2014b). The bioreactor chamber holds the scaffold in place and ensures hermetic sealing as well as force transmission onto the cell loaded construct. It consists of medium inlets/outlets, flow distributors, a flexible force transmitting disk, and the intended space for scaffold/construct placement. The power transmission rack includes a plunger, a pre-load screw, and a cam-shaft. The chamber is placed on the plunger and fixed via tightening of the pre-load screw. The cam-shaft moves the plunger in order to apply a sinusoidal compression pattern onto the bioreactor chamber.

To minimize the potential for contamination, all electronics and mechanical instrumentation were housed beneath the bioreactor chambers in a closed environment. The overall dimensions were kept sufficiently small (width × height × depth: 9 cm × 25 cm × 35 cm) to fit in a standard CO_2_ incubator (Figure 1C).

The system is controlled and monitored using a custom-made program based on LabView (NI, 622X, Austin, TX, USA) installed on a dedicated PC. This software controls the motor connected to the cam-shaft (Figure 1D) via a belt-drive generating a sinusoidal waveform for compression and simultaneously monitors data from four independent force sensors (Figure 1D, FC23 Compression Load Cell^®^, Measurement Specialties, VA, USA).

### Cell culture

Human mesenchymal stromal cells (MSC) were isolated from bone marrow aspirates (Braccini et al., 2005), after informed patient consent and following protocol approval by the local ethical committee (University Hospital Basel; Prof. Dr. Kummer; approval date 26/03/2007 Ref Number 78/07), and cultured as previously described (Frank et al., 2002). MSC were expanded for two to four passages for subsequent experiments. Then MSC were seeded on type I collagen-based OPTIMAIX scaffolds (O3D304030 Matricel, Germany, punched to cylindrical shape, 3 mm height, 8 mm diameter) or NiTi scaffolds (4 mm height, 8 mm diameter, open-porous 3D-printed structure) (Hoffmann et al., 2014a) with a seeding density of 4E + 06 cells/scaffold using a previously developed perfusion bioreactor system (Wendt et al., 2003, 2006). During the seeding phase, bidirectional perfusion was performed using syringe pumps at a perfusion velocity of 3 mL/min.

Open-porous, metallic NiTi scaffolds were included for the initial validation to ensure the broad operational range of the PCB. Further investigations were conducted using OPTIMAIX due to ease of histological assessments.

### Chondrogenic construct culture

Mesenchymal stromal cell seeded constructs were cultured for 3 weeks in chondrogenic medium (serum free medium supplemented with 0.1 mM ascorbic acid 2-phosphate, 10 ng/mL TGF-β3 and 10^−7^ M dexamethasone) (Mackay et al., 1998; Barbero et al., 2003). The serum free medium consists of: Dulbecco’s modified Eagle’s medium (DMEM) supplemented with 1 mM sodium pyruvate, 100 mM HEPES buffer, 100 U/mL penicillin, 100 μg/mL streptomycin, 0.29 mg/mL l-glutamine, ITS (10 μg/mL insulin, 5.5 μg/mL transferrin, 5 ng/mL selenium), 0.5 mg/mL bovine serum albumin, and 4.7 μg/mL linoleic acid. Using peristaltic pumps, unidirectional perfusion was applied to constructs with a perfusion velocity of 0.3 mL/min, with media changes twice per week (Santoro et al., 2011).

### Hypertrophic construct culture

In order to withstand dynamic mechanical loading, stable cartilaginous templates are a prerequisite. Therefore, only constructs exhibiting good chondrogenesis were included for further investigations. Following 3 weeks of culture, cartilaginous constructs were then separated in two groups: loaded and non-loaded. Non-loaded specimens were maintained in the unidirectional perfusion bioreactor whereas loaded specimens were transferred to the PCB system. Hypertrophic differentiation was induced by culturing constructs for 2 weeks in serum free medium supplemented with 50 nM thyroxine, 10 mM β-glycerophosphate, 10^−8^ M dexamethasone, and 0.1 mM l-ascorbic acid-2-phosphate (Scotti et al., 2010). Loaded constructs were exposed to an intermittent loading regime (construct displacement Δ*z* = 100 μm, frequency of *f*  = 1 Hz, three load cycles per day comprising 2 h of loading and 6 h of rest) for 2 weeks with a pre-load ensuring press fit of the construct. During the application of mechanical load, the applied forces were monitored for each bioreactor chamber separately.

### Quantification of glycosaminoglycan and DNA contents

Chondrogenic constructs were digested in proteinase K (1 mg/mL proteinase K in 50 mM Tris with 1 mM EDTA, 1 mM iodoacetamide, and 10 mg/mL pepstatin A) at 56°C overnight. The glycosaminoglycan (GAG) content of the cartilaginous constructs was determined spectrophotometrically using dimethylmethylene blue, with chondroitin sulfate as standard (Farndale et al., 1986). The DNA content of the constructs was measured using the CyQuant cell proliferation assay kit (Molecular Probes, Eugene, OR, USA) and used to normalize the GAG content.

### Real-time RT-PCR quantitation of transcript levels

Total RNA was extracted from cells using Trizol^®^ (LuBioScience GmbH, Lucerne, Switzerland) and reverse-transcribed as previously described (Frank et al., 2002). The samples were analyzed using a GeneAmp^®^ PCR System 9600 (Perkin Elmer, www.perkinelmer.com) and the transcription levels of the following genes of interest were quantified: collagen type-I, collagen type-II, aggrecan, cartilage oligomeric matrix protein (COMP), SOX9, and glyceraldehyde 3-phosphate dehydrogenase (GAPDH) as housekeeping gene (Barbero et al., 2003).

### Histology and immunohistochemistry

After *in vitro* cultures, the constructs were fixed in 1.5% paraformaldehyde and embedded in paraffin. Sections (5–10 μm thick) were stained for Safranin-O (Fluka) and Alizarin red after rehydration. Immunohistochemical analyses were performed to characterize the extracellular matrix using the following antibodies: collagen type-II (Col II; MPBiomedicals), collagen type-X (Col X; AbCam), and bone sialoprotein (BSP, 1:2000, A4232.1/A 4232.2, Immundiagnostik AG, Germany). Upon rehydration in ethanol series, sections were treated as previously described for antigen retrieval for Col II and Col X (Dickhut et al., 2009). The immunobinding was detected with biotinylated secondary antibodies and the appropriate Vectastain ABC kits. The red signal was developed with the Vector^®^ Red kit (Linaris AK-5000) and sections counterstained by Hematoxylin. Negative controls were performed during each analysis by omitting the primary antibodies. Histological and immunohistochemical sections were analyzed using an Olympus BX-63 microscope.

## Results

### Perfused compression bioreactor system

**Figure 2:**
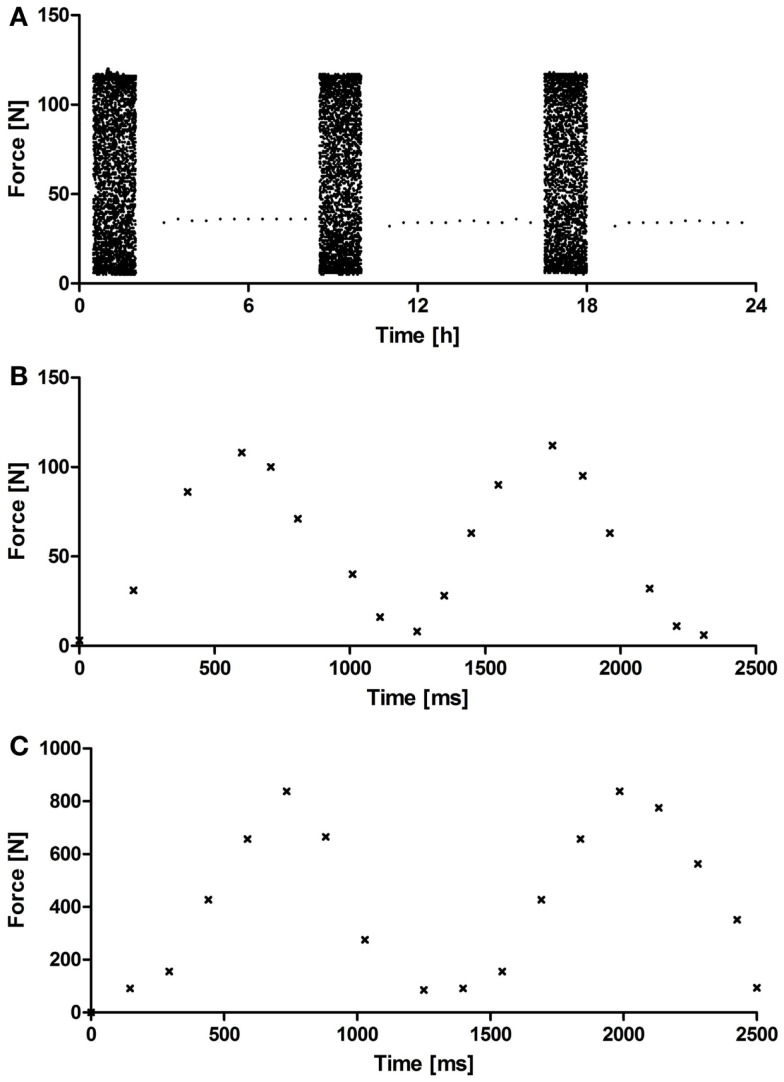
**Representative acquisition diagram from a force sensor**. **(A)** Representative force diagram acquired during 24 h of mechanical loading showing loading (2 h) and resting phases (4 h) for OPTIMAIX-based constructs (*n* = 4). Representative force diagram acquired during 2.5 s of loading showing the frequency (1 Hz) and periodicity of the sinusoidal wave for **(B)** OPTIMAIX- and **(C)** NiTi-based constructs (*n* = 4).

The custom-made PCB system (Figure 1) underwent a systematic validation of the cyclic compression regime and the monitoring of the force sensors over a period of 5 weeks. Figure 2 depicts representative force diagrams for chondrogenic constructs cultured under mechanical loading for 2 weeks. Figure 2A displays a force diagram of the daily loading regime consisting of loading (2 h) and resting phases (6 h). The force necessary to compress the chondrogenic constructs remained relatively constant throughout the entire culture period (Figure 2A). During the loading phase (Figures 2B,C), a sinusoidal waveform can be seen with a periodicity of approximately 1 s leading to the targeted frequency of 1 Hz. Moreover, the PCB showed a broad operational range as both collagen-based constructs (maximal force applied 110 N, Figure 2B) and NiTi-based constructs (maximal force applied 900 N, Figure 2C) could be stimulated without further modifications of the system.

### Chondrogenic differentiation

**Figure 3:**
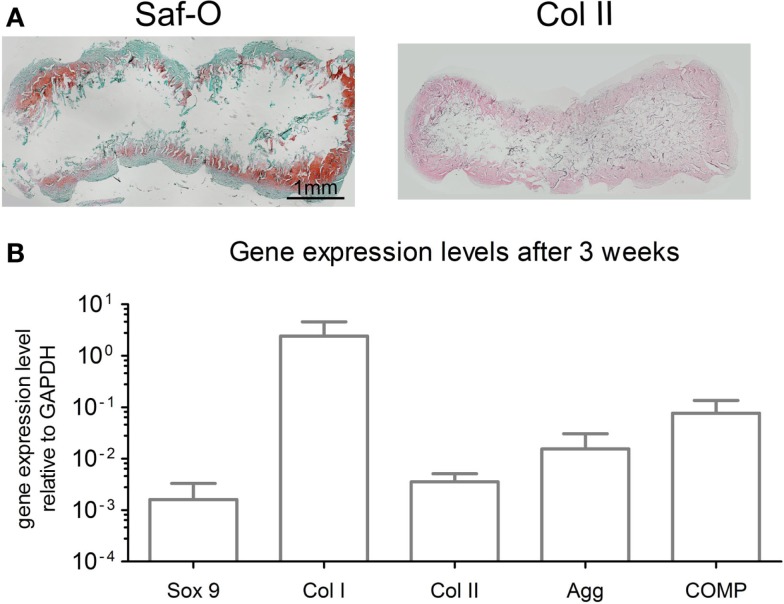
**Characterization of chondrogenic OPTIMAIX constructs (post 3 weeks of perfusion culture)**. **(A)** Representative histological Safranin-O staining for GAG and immunohistochemical staining for type-II collagen indicating cartilaginous tissue formation (*n* = 4). Scale bar = 1 mm (valid for both panels). **(B)** Gene expression levels for MSC cultured on scaffolds for 3 weeks. Measurements are mean ± SD (*n* = 3).

After 3 weeks of chondrogenic culture, MSC cultured on OPTIMAIX scaffolds could generate cartilaginous tissues. Cells were embedded in lacunae and deposited extracellular matrix positively stained for GAG and collagen type II (Figure 3A). GAG contents were determined to be 14.6 ± 3.4 μg/μg (GAG/DNA). Figure 3B shows the expression levels of genes associated with chondrogenic differentiation.

### Hypertrophic differentiation

**Figure 4:**
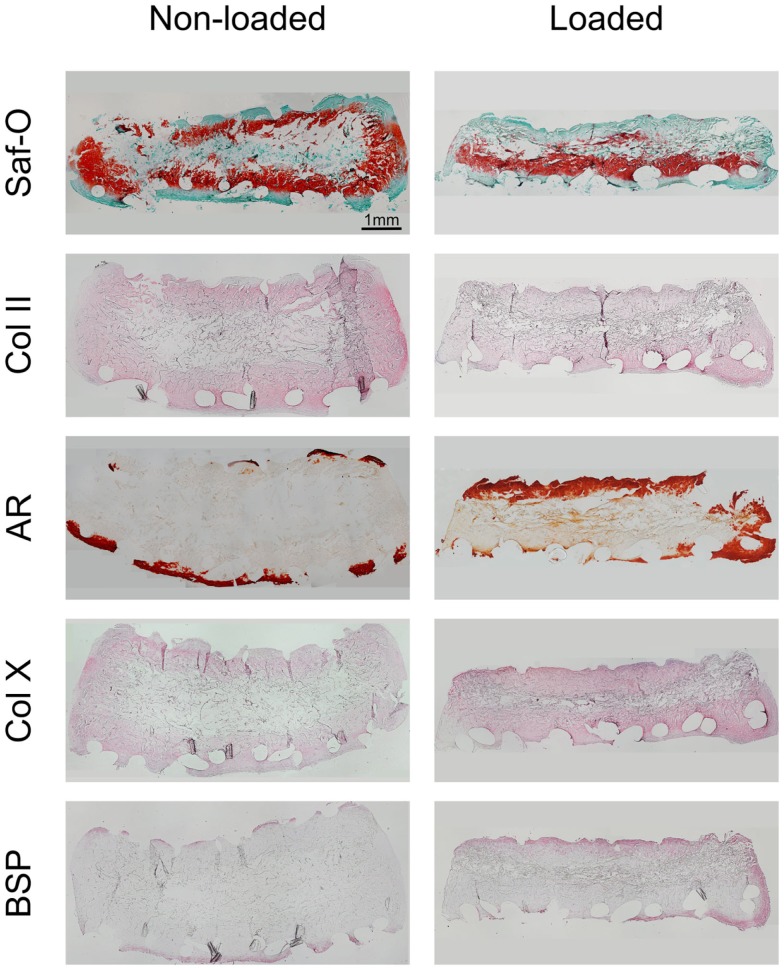
**Characterization of loaded and non-loaded constructs following the hypertrophic culture phase**. The panels depict histological Safranin-O staining, immunohistochemical staining for collagen type-II, alizarin red (AR) staining and immunohistochemical staining for collagen type-X, and bone sialoprotein (BSP), respectively (*n* = 4). Loaded constructs show a higher degree of maturation as compared to non-loaded constructs. Scale bar = 1 mm (valid for all panels).

Following 2 weeks of hypertrophic differentiation, constructs exhibited increased GAG deposition (Figure 4). Similar to chondrogenic constructs, the scaffold cores of non-loaded hypertrophic constructs were devoid of GAG, yet containing fibrotic tissue and limited amounts of cells. Loaded constructs exhibited a more homogeneous distribution of GAG, but less intense staining than non-loaded constructs indicating ongoing remodeling. Collagen type-II staining of non-loaded hypertrophically differentiated constructs was increased as compared to chondrogenic constructs and loaded hypertrophic constructs.

Alizarin red staining showed that the ECM of both loaded and non-loaded constructs was mineralized preferentially along the scaffold periphery. However, loaded constructs exhibited thicker mineralized borders as compared to non-loaded constructs as well as mineralized islets within the construct. Collagen type-X staining was also observed to be preferentially deposited at the scaffold periphery in both loaded and non-loaded constructs. However, it was enriched throughout the construct in loaded samples, especially in the highly mineralized regions. BSP immunohistochemical staining indicated relatively few positively stained cells in the periphery of the non-loaded scaffolds. Loaded constructs showed high amounts of BSP-positive cells in both the peripheral regions of the constructs and within the internal central region.

## Discussion

In this work, we have developed a PCB system to apply physiological dynamic mechanical loads and strains on engineered constructs to investigate the process of fracture healing. A proof-of-concept study was performed applying dynamic mechanical loads onto cell seeded collagen- and NiTi-based constructs, presenting the broad operational range of the developed system.

The PCB underwent systematic validation revealing safe and reliable functionality to ensure defined dynamic mechanical loading of viable engineered tissues. As compared to previously described systems (Rath et al., 2008; Ballyns and Bonassar, 2010; Sittichokechaiwut et al., 2010; Sun et al., 2010; Lujan et al., 2011; Matziolis et al., 2011; Omata et al., 2012; Petri et al., 2012; Puetzer et al., 2012; Shahin and Doran, 2012), the PCB exhibits a broader operational range. It allows for physiological mechanical compression in a consistent and reliable manner in a range from approximately 10 N up to 1 kN, while maintaining compact dimensions.

Given the fact that the compression applied is displacement driven, a various range of scaffolds and tissues can be easily investigated with dynamic mechanical loading without further adaptation of the system. Additionally, this range can be further enlarged via exchanging the eccentric cam-shaft, thereby adapting the displacement toward the desired range.

Our proof-of-concept study was conducted to assess the effect of physiological compressive loads during hypertrophic differentiation as an *in vitro* model for the transition from a soft to a hard callus. Since the development of a soft cartilaginous callus is a crucial step during secondary fracture healing (Schindeler et al., 2008), during the initial phase of the study, MSC were seeded on OPTIMAIX scaffolds and primed toward chondrogenesis. The resulting engineered constructs showed cartilaginous characteristics including: (i) ECM containing GAGs and collagen type-II, (ii) cells embedded in lacunae, and (iii) chondrocytic gene expression. Moreover, the cartilaginous constructs exhibited stable size and shape, enabling the application of dynamic loading within the PCB.

In our experimental setup, constructs undergoing mechanical loading during hypertrophy exhibited a higher degree of maturation than unloaded constructs. MSC embedded in the cartilaginous extracellular matrix of mechanically loaded constructs displayed enlarged lacunae to a higher extent than in non-loaded constructs. Furthermore, the diminished GAG and collagen type-II staining, as well as the high degree of mineral deposition (Mackie et al., 2008), collagen type-X content (Mackie et al., 2008; Gawlitta et al., 2010), and BSP staining (Sommer et al., 1996; Gawlitta et al., 2010) within loaded constructs underlines the late hypertrophic state of the MSC. Moreover, as BSP has been shown to be the main nucleator of hydroxyapatite crystals and to correlate with the initial phase of matrix mineralization (Bianco et al., 1993), the increased BSP staining in the ECM of mechanically loaded constructs indicates a higher degree of maturation as compared to non-loaded constructs. While this proof-of-concept study provides evidence of loading mediated hypertrophic differentiation, subsequent work should be aimed at further understanding the extent of hypertrophy (e.g., gene expression profiles).

In this study, we present our mechanically loaded hypertrophic constructs as an *in vitro* model of a fracture callus, which is undergoing the transition from soft to hard callus through remodeling and ossification of the soft cartilaginous callus (Gerstenfeld et al., 2003). The results obtained from our *in vitro* model system, i.e., application of dynamic mechanical compression during the hypertrophic differentiation phase, are consistent with previous *in vivo* models (Grundnes and Reikerås, 1991; Buckwalter and Grodzinsky, 1999; Hardy, 2004), which demonstrated that early mobilization and application of mechanical loads enhances the process of fracture healing. These results thus support the use of the PCB as an *in vitro* model for dynamic mechanical loading.

## Conclusion

In this study, we have demonstrated that the developed PCB system depicts a versatile tool for the *in vitro* application of dynamic physiological mechanical loads onto scaffolding materials with a wide range of mechanical properties. Mechanical loading applied via the developed bioreactor system enhances ECM mineralization during hypertrophy of cartilaginous constructs and triggers the maturation process of MSC toward late hypertrophic chondrocytes as demonstrated through the decrease in GAG and collagen type-II, the thickened mineralized border, the increased amounts of type-X collagen and positive BSP staining. Furthermore, the application of cyclic mechanical loading leads to the maturation of scaffold-based constructs. In combination with the fracture callus model, the PCB displays an advanced *in vitro* model and a promising tool for further studies testing alternative fracture treatments, based on engineered grafts or pharmaceutical compounds. Additionally, toward implementation of the 3R principles (replace, reduce, and refine) (Goldberg et al., 1996), this system could lead to a reduction of animal experiments within the field.

## Conflict of Interest Statement

The authors declare that the research was conducted in the absence of any commercial or financial relationships that could be construed as a potential conflict of interest.

## Supplementary Material

The Supplementary Material for this article can be found online at http://www.frontiersin.org/Journal/10.3389/fbioe.2015.00010/abstract
