# Supplementary material for: Novel Perfused Compression Bioreactor System as an in vitro Model to Investigate Fracture Healing
Source: Front Bioeng Biotechnol. 2015 Feb 2;3:10. doi: 10.3389/fbioe.2015.00010 (PMC4313709; doi:10.3389/fbioe.2015.00010)
Supplement: Supplementary file 1 [file Image_1.PDF]

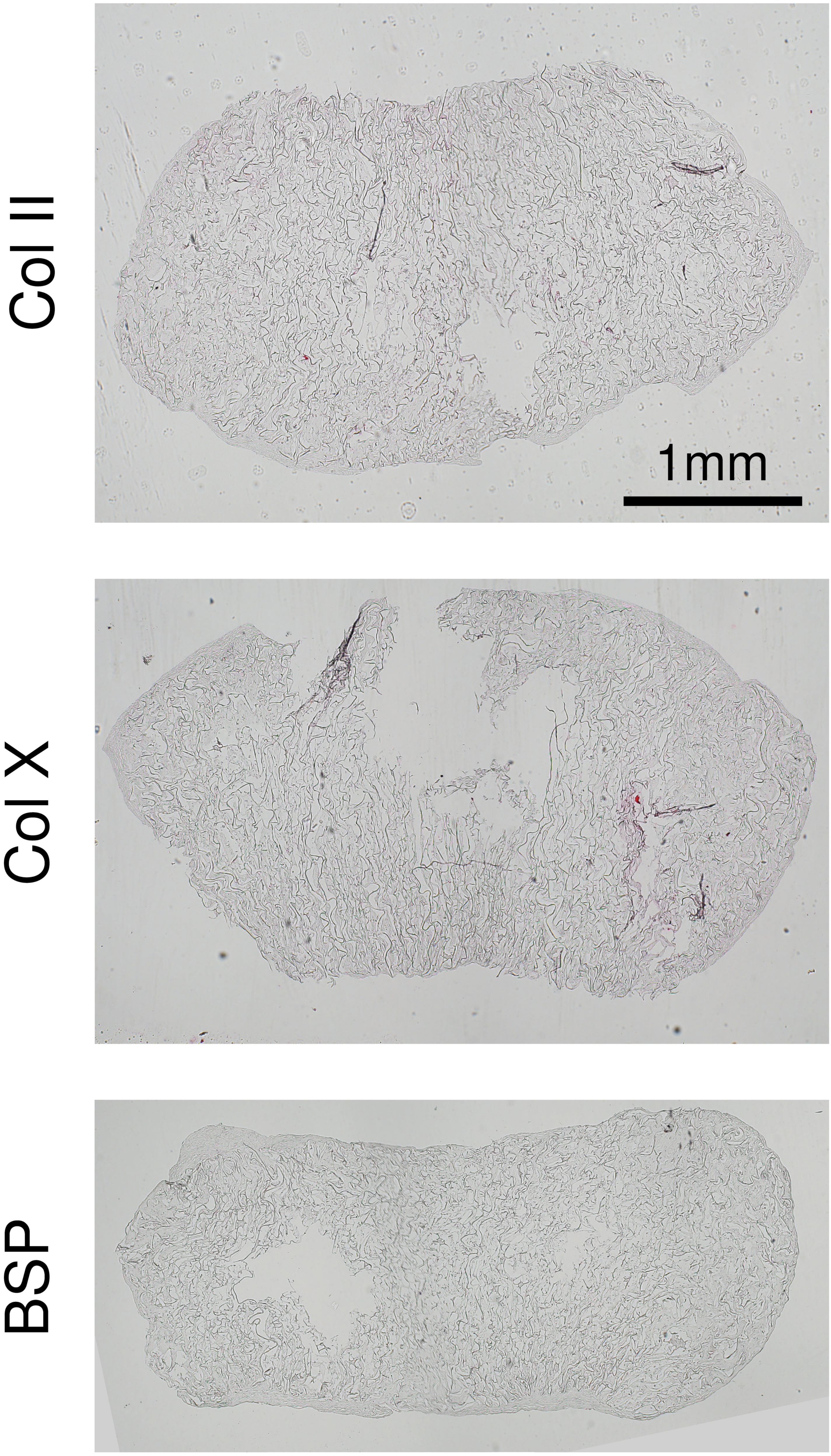

Supplementary Figure 1. Negative controls for immuno-histochemistry following the hypertrophic culture phase. The images show immunohistochemical staining for collagen type II, collagen type X and bone sialoprotein (BSP). The controls were performed omitting the secondary antibodies.
